# Clinical characteristics and prognosis of pneumonia-related bloodstream infections in the intensive care unit: a single-center retrospective study

**DOI:** 10.3389/fpubh.2023.1249695

**Published:** 2023-09-08

**Authors:** Yijie Liu, Ting Sun, Ying Cai, Tianshu Zhai, Linna Huang, Qi Zhang, Chunlei Wang, He Chen, Xu Huang, Min Li, Jingen Xia, Sichao Gu, Lingxi Guo, Bin Yang, Xiaojing Wu, Binghuai Lu, Qingyuan Zhan

**Affiliations:** ^1^Graduate School, Peking Union Medical College, Chinese Academy of Medical Sciences, Beijing, China; ^2^National Center for Respiratory Medicine, China-Japan Friendship Hospital, Beijing, China; ^3^State Key Laboratory of Respiratory Health and Multimorbidity, China-Japan Friendship Hospital, Beijing, China; ^4^National Clinical Research Center for Respiratory Diseases, China-Japan Friendship Hospital, Beijing, China; ^5^Institute of Respiratory Medicine, Chinese Academy of Medical Sciences, China-Japan Friendship Hospital, Beijing, China; ^6^Department of Pulmonary and Critical Care Medicine, Center of Respiratory Medicine, China-Japan Friendship Hospital, Beijing, China; ^7^Department of Radiology, China-Japan Friendship Hospital, Beijing, China; ^8^Vision Medicals Center for Infection Diseases, Guangzhou, China; ^9^Department of Pulmonary and Critical Care Medicine, The First Affiliated Hospital of Nanchang University, Nanchang University, Jiangxi, China

**Keywords:** bloodstream infections, pneumonia-related bloodstream infections, clinical characteristics, intensive care unit, gram-negative bacteria

## Abstract

**Background:**

Bloodstream infections (BSI) are one of the most severe healthcare-associated infections in intensive care units (ICU). However, there are few studies on pneumonia-related BSI (PRBSI) in the ICU. This study aimed to investigate the clinical and prognostic characteristics of patients with PRBSI in the ICU and to provide a clinical basis for early clinical identification.

**Methods:**

We retrospectively collected data from patients with bacterial BSI in a single-center ICU between January 1, 2017, and August 31, 2020. Clinical diagnosis combined with whole-genome sequencing (WGS) was used to clarify the diagnosis of PRBSI, and patients with PRBSI and non-PRBSI were analyzed for clinical features, prognosis, imaging presentation, and distribution of pathogenic microorganisms.

**Results:**

Of the 2,240 patients admitted to the MICU, 120 with bacterial BSI were included in this study. Thirty-two (26.7%) patients were identified as having PRBSI based on the clinical diagnosis combined with WGS. Compared to patients without PRBSI, those with PRBSI had higher 28-day mortality (81.3 vs.51.1%, *p* = 0.003), a higher total mortality rate (93.8 vs. 64.8%, *p* = 0.002), longer duration of invasive mechanical ventilation (median 16 vs. 6 days, *p* = 0.037), and prolonged duration of ICU stay (median 21 vs. 10 days, *p* = 0.004). There were no differences in other baseline data between the two groups, but patients with PRBSI had extensive consolidation on chest radiographs and significantly higher Radiographic Assessment of Lung Edema scores (mean 35 vs. 24, *p* < 0.001). The most common causative organisms isolated in the PRBSI group were gram-negative bacteria (*n* = 31, 96.9%), with carbapenem-resistant gram-negative bacteria accounting for 68.8% (*n* = 22) and multidrug-resistant bacteria accounting for 81.3% (*n* = 26).

**Conclusion:**

Pneumonia-related BSI is an important component of ICU-BSI and has a poor prognosis. Compared to non-PRBSI, patients with PRBSI do not have typical clinical features but have more severe lung consolidation lesions, and should be alerted to the possibility of their occurrence when combined with pulmonary gram-negative bacterial infections, especially carbapenem-resistant bacteria. Further multicenter, large-sample studies are needed to identify the risk factors for the development of PRBSI and prevention and treatment strategies.

## 1. Introduction

Bloodstream infections (BSI) are systemic infections caused by the invasion of pathogenic microorganisms and their toxins into blood circulation, which can lead to bacteremia, sepsis, and even death from septic shock (1–5). BSI often occurs in critically ill patients in intensive care units (ICU), especially those with mechanical ventilation with tracheal intubation, high inflammatory markers, immunosuppression, central venous line placement, extracorporeal membrane oxygenation (ECMO), and other invasive procedures (6, 7). Primary BSIs often do not have a clear primary source, and most BSIs are secondary, which can originate from intravascular catheters, abdominal infections, urinary tract infections, and pneumonia (8, 9). Appropriate antimicrobial therapy and early control of the source of infection are critical factors for reducing the morbidity and mortality associated with BSI. As one of the most lethal diseases, studies have revealed that the estimated mortality rate of BSI is up to 40% (10–12). Therefore, summarizing the clinical features of patients with BSI for accurate diagnosis and early intervention is important for reducing mortality and improving prognosis.

Pneumonia is one of the most frequent causes of secondary BSI in the ICU. In an international EUROBACT-2 study that included 2,600 ICU-BSI patients from 333 ICUs in 52 countries, pneumonia was the most frequent source of infection, and one study found a higher risk of death when pneumonia was the source of ICU-BSI (2, 10). However, the following problems still exist in the diagnosis of pneumonia-related bloodstream infection (PRBSI) in clinical practice: (1) critically ill patients in the ICU are often combined with disruption of the immune barrier; thus, the source of the causative bacteria may originate from multiple regions, making it difficult to accurately determine the primary lesion promptly; (2) clinicians can only make the clinical diagnosis of PRBSI based on pathogen culture results and time of onset, making it difficult to accurately identify patients with true PRBSI; and (3) because of the difficulty in diagnosis, few studies have reported the clinical features of PRBSI in the ICU, which seriously affects our understanding, early clinical diagnosis, and treatment of this category of secondary BSI.

Therefore, to further clarify the clinical features of PRBSI, we conducted a retrospective cohort study in the ICU of a tertiary care hospital to verify and confirm the clinical diagnosis of PRBSI by whole-genome sequencing (WGS) methods and to describe the clinical and prognostic features of PRBSI by collecting and analyzing clinical data of PRBSI patients to provide a clinical basis for early clinical recognition of PRBSI.

## 2. Materials and methods

### 2.1. Study design and patients

This single-center retrospective cohort study was conducted in the respiratory ICU (RICU) of the China-Japan Friendship Hospital in Beijing, China. We reviewed the clinical data of patients whose blood culture results were obtained during ICU admission between January 1, 2017, and August 31, 2020. The inclusion criteria were as follows: (1) age ≥ 18 years, (2) ICU stay ≥2 consecutive days, (3) positive peripheral blood culture results and blood cultures performed with at least two blood samples from multiple sites simultaneously, and (4) clinical features consistent with bloodstream infection. The exclusion criteria were as follows: (1) patients with blood culture results for fungi only and those considered to represent commensals or contaminants and (2) patients with incomplete medical records. In patients with more than one episode of BSI, only the first episode was considered and analyzed.

This study was approved (No. 2019-80-K52-1) by the institutional review board of the China-Japan Friendship Hospital, and the requirement for informed consent was waived. Patient data were anonymized before being used for analysis.

### 2.2. Data collection

Clinical data were reviewed using electronic medical record systems, and data of patients discharged from the hospital within 28 days were investigated via subsequent telephone follow-up. Data extracted included demographics [age, sex, body mass index (BMI), smoking status, and comorbidities (hypertension, chronic cardiovascular disease, chronic lung disease, brain disease, diabetes mellitus, immunosuppression, and malignancy)], basic vital signs, laboratory parameters, chest radiographic data, PaO_2_/FiO_2_ ratio, and basic and advanced respiratory support. Acute Physiology and Chronic Health Evaluation (APACHE) II and Sequential Sepsis-related Organ Function Assessment (SOFA) scores were assessed (13, 14) at ICU admission, 7 days before BSI, 72 h before BSI, 48 h before BSI, 24 h before BSI, and BSI onset, as well as outcome data, including duration of invasive mechanical ventilation (IMV), length of hospital stay, length of ICU stay, 28-day mortality, and total mortality. If several values for the same measured item were obtained during this period, the most abnormal values were recorded.

### 2.3. Definitions and microbiology methods

The clinical BSI cases were diagnosed by clinicians according to the criteria of the Centers for Disease Control and Prevention (CDC) (15). Clinical PRBSI was defined using three items: (1) if the microorganisms isolated from blood and respiratory samples, including sputum/bronchoalveolar lavage (BAL), shared the same species and drug resistance panels; (2) a sputum/BAL sample culture was performed within the infection window (3 days before and after the first positive blood culture); and (3) the clinical diagnostic criteria for pneumonia were met, which were based on the CDC guidelines.^1^ Patients considered to have combined bloodstream infections secondary to other primary infection sources were excluded. Subsequently, we performed WGS and phylogenetic analyses of the bacterial strains isolated from blood and respiratory tract samples of clinically diagnosed PRBSI patients. All analyses of raw sequencing data and interpretation of phylogenetic analyses were performed under the guidance of an experienced microbiologist. True PRBSI cases were identified in combination with clinical diagnosis and WGS. ICU-onset BSI is defined as positive culture results obtained using blood samples collected between ICU day three and ICU discharge (16).

Bronchoalveolar lavage or blood samples were collected based on standard clinical procedures. Microorganism cultures were processed using a Bactec-9240 system (Becton Dickinson, Sparks, MD, United States) or BacT/Alert 3D system (bioMérieux Inc., Marcy l’Etoile, France). The strains were identified based on colony morphology and MALDI-TOF MS (Bruker Daltonik, Bremen, Germany), according to the manufacturer’s recommendations. Antimicrobial susceptibility testing was conducted using the Vitek II automated system (BioMérieux, Inc.). Blood cultures were excluded if they were considered to represent commensals or contaminants, including common skin organisms and common colonizers in ICU patients (*coagulase-negative Staphylococci*, *Corynebacterium* spp., and *Cutibacterium* spp.). Multidrug-resistant (MDR) bacteria were defined as those resistant to three or more antimicrobial classes according to the criteria of the European Society of Clinical Microbiology and Infectious Diseases (ESCMID) (17). CR-NGRs were defined as isolates of gram-negative rods (NGRs) that were resistant to imipenem or meropenem. High-level aminoglycoside resistance was defined as MICs against gentamicin and amikacin both higher than 512 μg/mL. Positive results for the same organism in the same sample type from the same individual patient were recorded only once.

### 2.4. Whole-genome sequencing and phylogenetic analysis

Whole-genome sequencing has been performed for these pathogens (18). Microbial genomic DNA was extracted using a Qick-start Protocol DNeasy Mini kit (QIAGEN, Hilden, Germany) following the manufacturer’s protocol. Approximately 10 μg of DNA from each strain was used to construct Illumina paired-end libraries with an average insertion length of 500 base pairs (bp). Libraries were sequenced on the Illumina NovaSeq 6000 platform (Illumina Inc., San Diego, CA, United States). The following reads were removed from the raw data using fast with default parameters. The final cleaned reads with over 100× genome coverage for each strain were assembled using the megaHIT v1.2.9. The phylogenic tree based on the single nucleotide polymorphism (SNP) strategy and core-genome multilocus sequence typing (cgMLST) was constructed using the BacWGSTdb server, as previously described. MLST was performed using the Oxford scheme and sequence types were assigned using the MLST database.^2^ We then performed a phylogenetic analysis and constructed a phylogenetic tree of all strains isolated from respiratory and blood samples of patients with PRBSI. Graphs were generated using agree in R to display the evolutionary relationships.

### 2.5. Chest radiography

The chest radiographs obtained within 72 h before BSI onset were reassessed to grade the severity of imaging features based on the Radiographic Assessment of Lung Edema (RALE) score (19). As shown in Supplementary Figure 1, to determine the RALE score, each radiograph was divided into four quadrants defined by the vertebral column and the first branch of the left main bronchus. Each quadrant was assigned a consolidation score from 0 to 4 based on the percentage of involvement of the opacified lung, and a density score from 1 to 3 was assigned to assess the overall density of alveolar opacities. These two scores were multiplied for each quadrant and added to obtain a total score (ranging between 0 and 48) for each radiograph. The rating was performed by an experienced radiologist who was provided with sample cases on how to use the RALE score to reduce inter-rater variability. The RALE scores were considered continuous variables and categorized as ordinal response variables.

### 2.6. Statistical analysis

No sample size calculations were performed *a priori* for this descriptive study. Data are shown as the number of patients (%) for categorical variables and median (IQR) for continuous variables with non-normal distribution or mean (standard deviation) for those with normal distribution. The chi-squared test or Fisher’s exact test was used for categorical variables, and the *t*-test or nonparametric Mann–Whitney test was used to detect significant differences between the PRBSI and non-PRBSI groups. All statistical tests were two-tailed, and a value of *p* < 0.05 was considered significant. Analyses were conducted using the R Statistical Software (version 4.1.2, R Foundation for Statistical Computing, Vienna, Austria) and SPSS Statistics version 26.0 (IBM, Armonk, New York, United States).

## 3. Results

**Figure 1:**
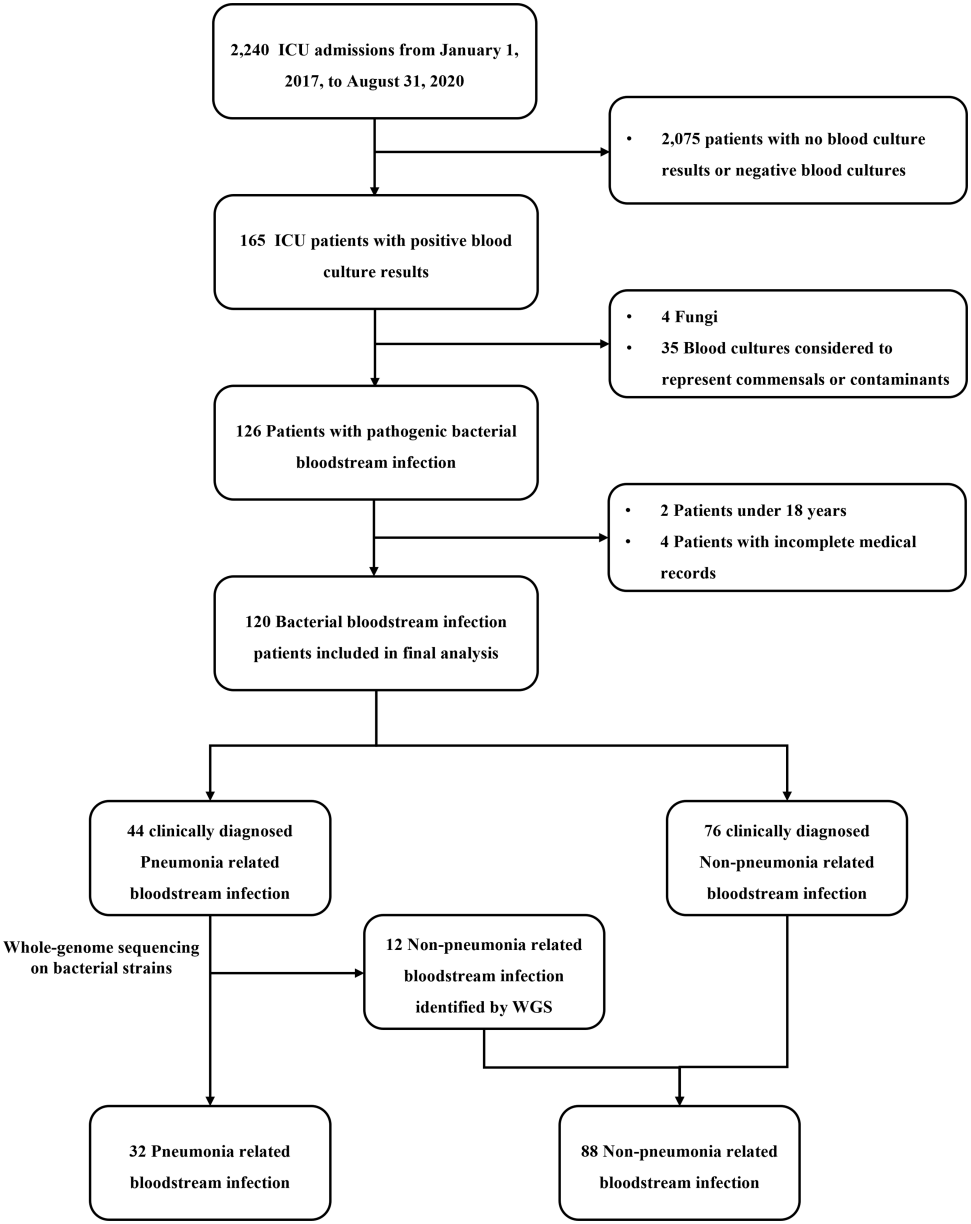
Flowchart of case selection in this study. ICU, intensive care unit; WGS, whole-genome sequencing.

Of the 2,240 ICU admissions, 120 (5.4%) consecutive patients were diagnosed with BSI between January 1, 2017, and August 31, 2020, and were included in the comparative analysis (Figure 1). Although the BSI source was not readily discernible, among the 120 ICU-BSI cases included, 37 (30.8%) cases were primary, 32 (26.7%) originated from pneumonia, and 28 (23.3%) were intravascular catheter-related (Supplementary Table 1). As shown in Figure 1, after screening all 2,240 patients with strict inclusion and exclusion criteria, we finally included 120 patients with BSI, 44 of whom were clinically diagnosed with PRBSI. After WGS of isolated bacterial strains from respiratory tract and blood specimens of all patients with clinically diagnosed PRBSI, we finally clarified that 32 of the patients identified by combination with clinical diagnosis and WGS as true PRBSI. Twelve cases with WGS detection of different bacterial strains and the 76 cases with clinically diagnosed non-PRBSI together formed the non-PRBSI group. We performed phylogenetic analysis for strains and conducted a phylogenetic tree based on the whole genome sequencing data (Supplementary Figure 2). It showed that the strains from the respiratory tract and blood were considered to be the same in 32 patients (including 14 *Pseudomonas aeruginosa,* 7 *Acinetobacter baumannii*, 5 *Klebsiella pneumoniae*, 2 *Burkholderia multivorans*, 1 *Escherichia coli*, 1 *Morganella morganii*, 1 *Burkholderia cenocepacia*, and 1 *Staphylococcus aureus*). Among the rest 12 patients, three specimens from the respiratory tract received contamination from other strains and nine were confirmed not to be 100% homologous. Finally, among 120 BSI patients, 32 (26.7%) patients were identified as having PRBSI *via* combined clinical and WGS analysis, whereas 88 (73.3%) were considered non-PRBSI.

### 3.1. Patient characteristics

Patient characteristics were compared between the patients with and without PRBSI (Table 1). There were no differences in the demographic characteristics between the two groups. The median age of the PRBSI group was 66 years, and 19 (59.4%) were male, while the non-PRBSI group was 65 years, and males accounted for 69.3%. The most common primary cause of ICU admission for patients in our ICU was respiratory disease (*n* = 96, 80.0%), especially pneumonia (*n* = 63, 52.5%). At the onset of BSI, fewer patients in the PRBSI group were exposed to antibiotics within 30 days prior to BSI (34.4 vs. 53.4%). The median onset of PRBSI was 13 days post-hospital admission and 12 days post-ICU admission, which was longer than that for those without PRBSI (median 5 vs. 3 days, *p* < 0.001). Twenty-eight (87.5%) patients had ICU-onset BSI, which was considered ICU-acquired, compared to only 51.1% of the non-PRBSI patients (*p* < 0.001). Twenty-six (81.3%) patients with PRBSI required invasive mechanical ventilation (IMV) before BSI, and the median duration of IMV was 9 days. The median APACHE II score was 20 and the mean SOFA score was 9 at the onset of BSI in the PRBSI group. Patients with PRBSI had worse outcomes. The mean length of ICU stay for patients with PRBSI was 21 days, which was significantly longer than the 10 days for patients without PRBSI (*p* = 0.004). The median number of days of advanced respiratory support (IMV) was 16 days for patients with PRBSI and longer than 6 days for patients without PRBSI (*p* = 0.037). According to electronic medical records and telephone follow-ups, the 28-day mortality rate was much higher in the PRBSI group (81.3 vs. 51.1%, *p* = 0.003). The total mortality rate was 93.8% in the PRBSI group and 64.8% in the non-PRBSI group (*p* = 0.002). Meanwhile, the curves of laboratory indicators related to infection, including white blood cell count, neutrophil count, platelet count, lactic acid, procalcitonin, and D-dimer levels, during the period from ICU admission to BSI diagnosis, are summarized (*p* > 0.05; Supplementary Figure 3; Supplementary Table 2). We found that PRBSI patients had significantly lower white blood cell count and neutrophil count than non-PRBSI patients on ICU admission, and there were no statistically significant differences between the PRBSI group and the non-PRBSI group at different time points prior to the onset of BSI for other infection-related indicators described above.

**Table 1:** Demographics, characteristics at BSI time, and outcomes for PRBSI and Non-PRBSI groups.

	Total BSI	PRBSI	Non-PRBSI	*p* value
	*n* = 120	*n* = 32	*n* = 88	
Demographics				
Age, years	65 (55, 73)	66 (54, 72)	65 (56, 74)	0.771
Male sex	80 (66.7%)	19 (59.4%)	61 (69.3%)	0.307
BMI	23.15 (20.78, 26.13)	21.55 (18.58, 25.33)	23.75 (21.48, 26.35)	0.092
Smoker	50 (40.1%)	11 (34.4%)	39 (44.3%)	0.329
Comorbidities				
Hypertension	54 (45.0%)	13 (40.6%)	41 (46.6%)	0.561
Chronic cardiovascular disease	34 (28.3%)	7 (21.9%)	27 (30.7%)	0.344
Chronic lung disease	33 (27.5%)	7 (21.9%)	26 (29.5%)	0.405
Brain disease	21 (17.5%)	4 (12.5%)	17 (19.4%)	0.385
Diabetes mellitus	33 (27.5%)	10 (31.3%)	23 (26.1%)	0.579
Immunosuppression	42 (35.0%)	15 (46.9%)	27 (30.7%)	0.100
Malignancy	16 (13.3%)	7 (21.9%)	9 (10.2%)	0.175
Primary cause for ICU admission				
Respiratory diseases	96 (80.0%)	29 (90.6%)	67 (76.1%)	0.079
Pneumonia	63 (52.5%)	16 (50.0%)	47 (53.4%)	0.741
Interstitial lung disease	16 (13.3%)	4 (12.5%)	12 (13.6%)	1.000
Other respiratory diseases	17 (14.2%)	9 (28.1%)	8 (9.1%)	0.008
Sepsis	8 (6.7%)	0 (0.0%)	8 (9.1%)	0.176
Cardiovascular diseases	7 (5.8%)	3 (9.4%)	4 (4.5%)	0.577
Abdominal diseases	6 (5.0%)	0 (0.0%)	6 (6.8%)	0.297
Other diseases	3 (2.5%)	0 (0.0%)	3 (3.4%)	0.564
Patient characteristics at BSI diagnosis				
Antibiotic exposure within 30 days before BSI	58 (48.3%)	11 (34.4%)	47 (53.4%)	0.065
Antibiotic exposure within 7 days before BSI	101 (84.2%)	29 (90.6%)	72 (81.8%)	0.243
Time from hospital admission to BSI, days	7 (1, 17)	13 (8, 22)	5 (1, 14)	0.001
Time from ICU admission to BSI, days	5 (1, 14)	12 (7, 21)	3 (1, 9)	<0.001
Requiring IMV before BSI	78 (65.0%)	26 (81.3%)	52 (59.1%)	0.024
IMV time before BSI, days	3.5 (0, 10)	9 (3, 16)	2 (0, 8)	0.002
ICU-onset BSI	73 (60.8%)	28 (87.5%)	45 (51.1%)	<0.001
APACHE II at BSI onset	19 (8)	20 (9)	18 (7)	0.391
SOFA at BSI onset	10 (4)	9 (4)	10 (4)	0.609
IMV at BSI onset	80 (67.8%)	22 (68.8%)	58 (67.4%)	0.892
P/F ratio, mmHg	145.50 (83.46, 211.36)	118.83 (76.43, 191.71)	150.33 (86.41, 218.67)	0.224
Noradrenalin at BSI onset	76 (63.3%)	18 (56.3%)	58 (65.9%)	0.332
Corticosteroids for sepsis or septic shock	82 (68.3%)	26 (81.3%)	56 (63.6%)	0.067
Respiratory tract co-infection				
Bacteria	90 (75.0%)	32 (100.0%)	58 (65.9%)	<0.001
Virus	61 (50.8%)	17 (53.1%)	44 (50.0%)	0.762
Fungi	60 (50.0%)	14 (43.8%)	46 (52.3%)	0.409
Outcomes				
Days of advanced respiratory support (IMV)	7 (2, 20)	16 (5, 25)	6 (0, 18)	0.037
Length of hospital stay, days	17 (8, 32)	21 (12, 31)	16 (7, 32)	0.209
Length of ICU stay, days	12.5 (6, 27)	21 (12, 31)	10 (6, 20)	0.004
28-day mortality	71 (59.2%)	26 (81.3%)	45 (51.1%)	0.003
Total mortality	87 (72.5%)	30 (93.8%)	57 (64.8%)	0.002

All continuous data are given as median (IQR) or mean (SD), all categorical data as proportion (%). BSI, Bloodstream infection; BMI, Body mass index; P/F ratio, PaO2/FiO2 ratio; APACHE-II, acute physiology and chronic health evaluation-II; SOFA, sequential organ failure assessment score; ICU, intensive care unit; and IMV, invasive mechanical ventilation.

### 3.2. Chest radiograph scoring and respiratory support analysis

**Figure 2:**
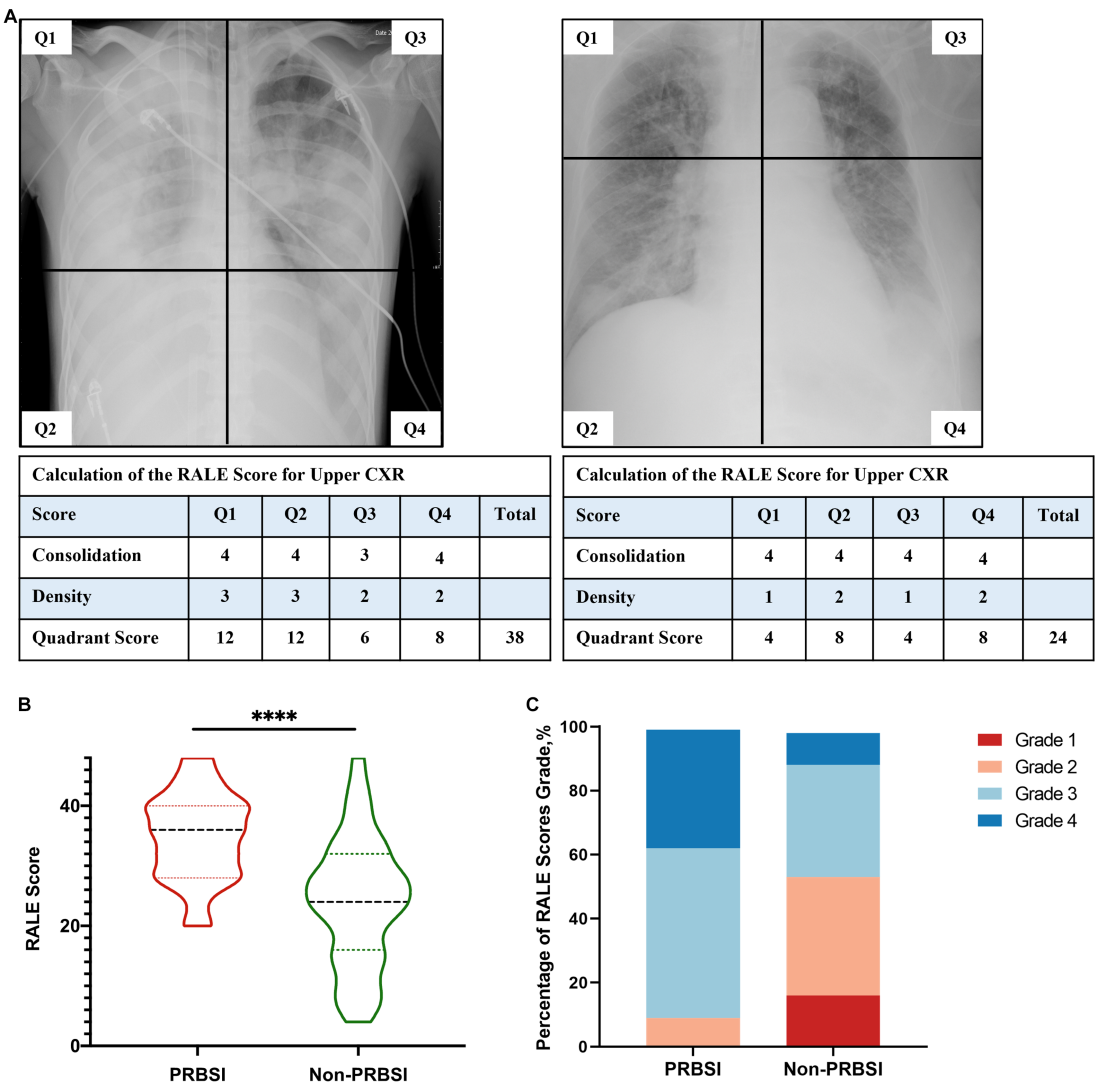
Examples of radiographic assessment of lung edema (RALE) score for pulmonary disease severity in our study **(A)**. PRBSI patients were more likely to have higher RALE scores and higher RALE score grades on chest radiographs within 72 h prior to BSI diagnosis, compared with non-PRBSI patients **(B,C)**.

To investigate the severity of chest imaging features, we conducted a RALE score assessment on chest radiographs within 72 h prior to BSI onset to evaluate the degree and extent of pulmonary consolidation (Table 2; Figure 2). A comparison of RALE scores revealed that patients with PRBSI had higher RALE scores (35 ± 7) than those without non-PRBSI patients (24 ± 11; *p* < 0.001). The grades were also significantly different between the two groups (*p* < 0.001). In the PRBSI group, 29 (90.6%) patients had a RALE score of >24. Simultaneously, we compared the IMV parameters within 72 h before BSI between the two groups and no significant differences were detected. Thirteen (40.6%) patients in the PRBSI group received prone position treatment, while only 19(22.4%) of the non-PRBSI patients received this treatment strategy (*p* = 0.048). Five (15.6%) and 10 (11.8%) patients with PRBSI underwent lung recruitment maneuvers, respectively (Table 2).

**Table 2:** Chest radiograph scoring and respiratory support analysis between PRBSI and non-PRBSI groups.

Characteristics	Total BSI	PRBSI	Non-PRBSI	*p* value
	*n* = 120	*n* = 32	*n* = 88	
RALE score within past 72h before BSI dignosis^a^	27 (11)	35 (7)	24 (11)	<0.001
RALE score grades				<0.001
1 (0–12)	14 (11.8%)	0 (0.0%)	14 (16.1%)	
2 (13–24)	36 (30.3%)	3 (9.4%)	33 (37.9%)	
3 (25–36)	48 (40.3%)	17 (53.1%)	31 (35.6%)	
4 (37–48)	21 (17.6%)	12 (37.5%)	9 (10.3%)	
Invasive mechanical ventilation parameters				
72 h before BSI				
PC/PS	15 (4)	15 (4)	15 (4)	0.880
PEEP	8 (6, 10)	8 (6, 12)	8 (5, 9.5)	0.574
FiO_2_	0.60 (0.24)	0.61 (0.27)	0.60 (0.23)	0.925
48 h before BSI				
PC/PS	14 (12, 18)	14 (12, 18)	15 (12, 17)	0.842
PEEP	8 (5, 10)	8 (6, 10.5)	7.5 (5, 8)	0.115
FiO_2_	0.59 (0.22)	0.60 (0.24)	0.59 (0.22)	0.818
24 h before BSI				
PC/PS	14.5 (12, 18)	14 (12, 16)	15 (12, 18)	0.222
PEEP	8 (5.5, 10)	8 (6, 10)	8 (5, 10)	0.655
FiO_2_	0.60 (0.44, 0.81)	0.52 (0.40, 0.77)	0.60 (0.45, 0.86)	0.239
BSI onset				
PC/PS	14 (12, 18)	14 (12, 18)	14 (12, 18)	0.990
PEEP	8 (5, 10)	9 (6, 10)	7.5 (5, 8.5)	0.218
FiO_2_	0.68 (0.50, 1.00)	0.70 (0.49, 1.00)	0.68 (0.50,1.00)	0.729
Prone position treatment	32 (27.4%)	13 (40.6%)	19 (22.4%)	0.048
Recruitment maneuver	15 (12.8%)	5 (15.6%)	10 (11.8%)	0.578

^a^When chest radiographic examinations were performed several times within 72 h before BSI onset, the worst result was recorded. One patient’s CRX data was not available. All continuous data are given as median (IQR) or mean (SD), all categorical data as proportion (%). BSI, Bloodstream infection; RALE, Radiographic assessment of lung edema; PC, Pressure control; PS, Pressure support; PEEP, Positive end-expiratory pressure.

### 3.3. Pathogens isolated from BSI patients

Among all 120 BSI patients included in the study, all 32 PRBSI patients had one bacterium cultured in their blood cultures, while 10 of the 88 non-PRBSI patients had a combination of two bacterial infections. Therefore, of the 130 organisms isolated from all patients with ICU BSI, Gram-negative bacteria were more prevalent, with a detection rate of 76.1% (*n* = 99), and gram-positive bacteria in 23.8% (*n* = 31; Table 3).The most common organism in the PRBSI group was *Pseudomonas aeruginosa* (14/32, 43.8%), followed by *Acinetobacter baumannii* (7/32, 21.9%) and *Klebsiella pneumoniae* (5/32, 15.6%; Supplementary Figure 4). A significantly different distribution of pathogens was observed between the PRBSI and non-PRBSI groups. Among the PRBSI group isolates, 31 (96.9%) were gram-negative bacteria and 22 (68.8%) were carbapenem-resistant bacteria, both of which had a significantly higher incidence than that in the non-PRBSI group (*p* < 0.01). The incidence rate of multidrug-resistant organism (MDRO) infection was similar in both groups, whereas that of gram-negative bacteria and gram-positive bacteria was significantly different (*p* < 0.01).

**Table 3:** Pathogens detection between PRBSI and non-PRBSI groups.

Pathogen	Total BSI	PRBSI	Non-PRBSI	*p* value
	no. (%)	no. (%)	no. (%)	
Total pathogens	130 (100)	32 (24.6)	98 (75.4)	…
Gram-negative bacteria	99 (76.1)	31 (96.9)	68 (69.4)	0.002
*Pseudomonas aeruginosa*	27 (20.8)	14 (43.8)	13 (13.3)	<0.001
*Acinetobacter baumannii*	21 (16.2)	7 (21.9)	14 (14.3)	0.311
*Klebsiella pneumoniae*	21 (16.2)	5 (15.6)	16 (16.3)	0.925
*Burkholderia multivorans*	3 (2.3)	2 (6.3)	1 (1.0)	0.150
*Burkholderia cenocepacia*	9 (6.9)	1 (3.1)	8 (8.2)	0.566
*Escherichia coli*	11 (8.5)	1 (3.1)	10 (10.2)	0.377
Other gram-negative bacteria	7 (5.4)	1 (3.1)	6 (6.1)	0.841
Carbapenem-resistant bacteria	48 (36.9)	22 (68.8)	26 (26.5)	<0.001
CRPA	22 (16.9)	13 (40.6)	9 (9.2)	<0.001
CRAB	17 (13.1)	7 (21.9)	10 (10.2)	0.162
CRE	9 (6.9)	2 (6.3)	7 (7.1)	1.000
Gram-positive bacteria	31 (23.8)	1 (3.1)	30 (29.9)	0.002
*Staphylococcus aureus*	8 (6.2)	1 (3.1)	7 (7.1)	0.691
*Enterococcus faecium*	16 (12.3)	0 (0.0)	16 (15.5)	0.033
*Enterococcus faecalis*	6 (4.6)	0 (0.0)	6 (6.1)	0.343
*Streptococcus pneumoniae*	1 (0.8)	0 (0.0)	1 (1.0)	1.000
HLAR	14 (10.8)	0 (0.0)	14 (14.3)	0.053
MDRO in all bacteria	92 (70.8)	26 (81.3)	66 (67.3)	0.133
MDRO in GNRs	48 (36.9)	26 (81.3)	42 (42.9)	<0.001
MDRO in GPRs	24 (18.5)	0 (0.0)	24 (24.5)	0.002

BSI, Bloodstream infection; CRPA, Carbapenem-resistant *Pseudomonas aeruginosa*; CRAB, Carbapenem-resistant *Acinetobacter baumannii*; CRE, Carbapenem-resistant *Enterobacteriaceae*; MDRO, Multidrug-resistant organism; GNRs, Gram-negative rods; GPRs, Gram-positive rods.

## 4. Discussion

To the best of our knowledge, this is the first study to identify and demonstrate the clinical characteristics of patients with PRBSI in the ICU. Our study innovatively used clinical diagnosis combined with WGS to identify patients with PRBSI. In the present study, there were several interesting findings: (1) most of the secondary BSI in our ICU was pneumonia-related; (2) compared with non-pneumonia BSI, PRBSI was associated with poor outcomes, including prolonged IMV duration, length of ICU stay, increased 28-day mortality, and total mortality in the ICU; (3) lung imaging findings in the PRBSI group showed more consolidation on chest radiographs; and (4) PRBSI displayed a different distribution of causative pathogens.

At present, most previous ICU-BSI studies have focused on intravascular catheter-related BSI, which has several evidence-based guidelines for prevention and reduction (20). However, very few studies have reported PRBSI. A retrospective study that included 141 cases of hospital-acquired *A. baumannii* bacteremia found that patients in the pneumonia-related group had a worse prognosis, higher rates of antibiotic resistance, and higher rates of complications than those without pneumonia (21). Another study that included 188 cases of *A. baumannii* bacteremia in the ICU compared the differences and risk factors for prognosis between patients with pneumonia- and non-pneumonia-related bacteremia and found that patients with pneumonia-related bacteremia had more carbapenem-resistant strains and a higher 30-day mortality rate (22). Both these studies only explored the characteristics of pneumonia-related *A. baumannii* bloodstream infections. However, different pathogenic microorganisms may have a significant impact on the clinical presentation and prognosis of BSI.

In this study, we identified PRBSI cases, combined with clinical diagnoses and WGS. In clinical practice, physicians diagnose BSI according to patients’ symptoms and organism culture tests and consider the most likely source. Our study used WGS and phylogenetic analysis to validate the same bacterial strains isolated from the blood and respiratory tract specimens. Previous studies have shown that pneumonia or respiratory tract infection is one of the most important sources of secondary BSI in the ICU (8, 10). Our study showed that the proportion of PRBSI in ICU-BSI was 26.7%, consistent with that in the EUROBACT-1 international study (21%), the EUROBACT-2 cohort study (26.7%), and a recent study that recruited 223 patients (25%) (10, 23, 24). Patients with PRBSI showed prolonged hospital and ICU stays prior to BSI onset, suggesting that BSI occurring early in ICU admission is more likely to originate from sources other than PRBSI.

Several studies have shown that BSI in the ICU is associated with poor clinical outcomes. The prolonged median length of ICU stay for ICU-acquired BSI varies from 9 to 13 days (24, 25) is consistent with our findings of 12 days. In a large ICU-BSI study (*n* = 571), the risk of death was 40%, and the adjusted hazard ratio was even higher when the source was pneumonia (2). In our study, patients with PRBSI showed significantly prolonged ICU stays for 21 days and a higher 28-day mortality rate (81.3%), and a total mortality rate (93.8%). However, laboratory indicators related to infection during the period from ICU admission to BSI diagnosis did not demonstrate any significant differences, suggesting that laboratory examinations may have a limited role in identifying PRBSI. PRBSI, which has high morbidity and a very poor prognosis in patients with ICU-BSI, should be given more attention in both clinical practice and research.

Early appropriate source control for secondary BSI is important to improve the condition and should be guided by the patient’s clinical presentation. The most common conditions that may require a specific approach for source control are vascular devices, skin and soft tissue, and intra-abdominal infections (6). For those that originated from pneumonia, extensive consolidation was normally observed in the patients’ chest radiographs prior to the onset of BSI. To assess the extent and density of pulmonary lesion involvement, we used the RALE score, which has been proven reliable in the assessment of opacification on COVID-19 patients’ chest radiographs on admission and helps predict adverse outcomes, to quantify the chest radiographic severity within 72 h before BSI diagnosis (26). A similar proportion of patients had pneumonia as the primary cause of ICU admissions, but there was a significant difference in chest radiograph RALE scores at 72 h prior to BSI diagnosis (*p* < 0.001), suggesting that chest radiography was positive for the identification of PRBSI. In our study, patients with PRBSI were more likely to present with increased disease severity from pneumonia on chest radiographs, indicating that the alveoli were more extensively affected by the infected foci. Among pneumonia patients with severe extensive consolidation, the alveoli were filled with a solidified mixture of bacteria and inflammatory cells, gas could not enter or exit, and alveolar collapse occurred. It is necessary to take measures to promote the drainage of secretions, reopen alveoli, and improve pulmonary ventilation. There are many approaches to decrease the amount of collapsed tissue and keep it open, including endotracheal or tracheostomy suctioning, high positive end-expiratory pressure (PEEP), prone position treatment, and lung recruitment maneuvers (27, 28). In a study of 1,057 COVID-19 patients who were intubated and mechanically ventilated, the prone position induced a significant increase in the PaO_2_/FiO_2_ ratio, increased ventilation-perfusion matching, and improved oxygenation (29). Another study of 99 COVID-19 patients on Extracorporeal Membrane Oxygenation (ECMO) demonstrated that the PEEP level is required to counteract compressive forces leading to lung collapse, and PEEP that increases from 5 to 15 cmH_2_O appears favorable (30). Therefore, for patients with PRBSI with extensive consolidation on chest radiographs, we suggest that it is necessary to adopt advanced strategies aimed at reopening collapsed or obstructed peripheral airways and alveoli and improving the primary infection lesions.

Another effective primary source of control in pneumonia-related BSI is early and appropriate antimicrobial treatment, in which recognizing the distribution of pathogens causing BSI and their antibiotic resistance is important. The causative pathogens of BSI can be severe types of microbes, including bacteria and fungi, of which bacteria are responsible for over 90% of all cases (31). In our study, Gram-negative bacteria were the most commonly isolated pathogens among critically ill patients with BSI (76.1%), especially in patients with PRBSI. *Pseudomonas aeruginosa* was most frequently isolated from the PRBSI group, followed by *A. baumannii* and *K. pneumoniae.* A study including patients with ICU-BSI over 12 years showed a significant increase in gram-negative bacterial infections (32). The EUROBACT International Cohort Study also underscored this pattern by showing that nearly 60% of BSI cases are caused by Gram-negative bacteria (23). In comparison, the EPIC III cohort study investigated the prevalence and outcomes of ICU patients with infections in 2017 and reported a higher proportion of gram-positive pathogens (11). Another concern is the high rate of drug-resistant pathogen detection. In our study, 70.8% of patients with ICU-BSI had MDRO infections and 36.9% of carbapenem-resistant gram-negative bacteria were isolated. Among patients with PRBSI, the incidence rates of MDRO (26/32, 81.3%) and carbapenem-resistant bacteria (22/32, 68.8%) were even higher and became dominant. Previous studies have revealed an increasing rate of antimicrobial resistance even in countries with low MDRO (23, 33, 34). Carbapenem resistance in gram-negative bloodstream infections is associated with mortality, highlighting the importance of preventing and treating infections caused by multidrug-resistant pathogens (10, 35, 36). Our results might help in the instantaneous selection of empiric antibiotic therapy for patients suspected of having pneumonia-related BSI.

However, this study had several limitations. First, it should be noted that our sample size was limited, and any related conclusions should be drawn cautiously. Therefore, the results of this study may not apply to other institutions. Another important limitation is that we performed a single-center retrospective study, mainly because of possible information and selection biases in tracing exposure history. Finally, the study was conducted in a tertiary hospital, to which many local hospitals transferred unexpectedly severely ill patients, and there might be a chance of selection bias. Further continuous and large-scale multicenter prospective studies on PRBSI are required to validate our findings.

## 5. Conclusion

Our study showed that PRBSI is associated with a high proportion of ICU-BSI and poor clinical outcomes. Compared to patients with non-PRBSI, PRBSI was more likely to be ICU-acquired, had more severe consolidation involvement on chest radiography, and was mainly caused by gram-negative bacteria, predominantly MDRO and carbapenem-resistant bacteria. These findings can be applied to the early recognition and management of patients with PRBSI in the ICU.

## Data availability statement

The datasets presented in this study can be found in online repositories. The names of the repository/repositories and accession number(s) can be found at: https://ngdc.cncb.ac.cn, PRJCA015244.

## Ethics statement

The studies involving humans were approved by institutional review board of the China-Japan Friendship Hospital. The studies were conducted in accordance with the local legislation and institutional requirements. Written informed consent for participation was not required from the participants or the participants’ legal guardians/next of kin in accordance with the national legislation and institutional requirements.

## Author contributions

YJL, TS, XJW, and QYZ contributed to the study conception and design. YJL and TS contributed to data acquisition. HC reassessed the chest radiographs. CLW and BHL provided an interpretation of microbiology. BY helped perform the WGS and phylogenetic analyses. YJL and XJW analyzed the data. YJL drafted the manuscript. All authors contributed to the article and approved the submitted version.

## Funding

This work was supported by the CAMS Innovation Fund for Medical Sciences (2022-I2M-JB-016) to QYZ, National High-Level Hospital Clinical Research Funding (2022-NHLHCRF-LX-01-01) to QYZ, the CAMS Innovation Fund for Medical Sciences (2021-I2M-1-030) to BHL, the Medical Talent Program for High-throughput Sequencing Technology in Infectious Diseases, China (Grant no. MTP2022A004) to XJW, the National High Level Hospital Clinical Research Funding—Elite Medical Professionals Project of China-Japan Friendship Hospital (NO.ZRJY2023-GG20) to XJW, and Key Research and Development Program of Jiangxi province (20232BBG7002) to XJW.

## Conflict of interest

The authors declare that the research was conducted in the absence of any commercial or financial relationships that could be construed as potential conflicts of interest.

## Publisher’s note

All claims expressed in this article are solely those of the authors and do not necessarily represent those of their affiliated organizations, or those of the publisher, the editors and the reviewers. Any product that may be evaluated in this article, or claim that may be made by its manufacturer, is not guaranteed or endorsed by the publisher.

## Footnotes

^1^ https://www.cdc.gov/nhsn/pdfs/pscmanual/4psc_clabscurrent.pdf

^2^ http://pubmlst.org/

## References

[1] Wisplinghoff H Bischoff T Tallent SM Seifert H Wenzel RP Edmond MB. Nosocomial bloodstream infections in US hospitals: analysis of 24,179 cases from a prospective nationwide surveillance study. Clin Infect Dis. (2004) 39:309–17. doi: 10.1086/421946, PMID: 15306996

[2] Adrie C Garrouste-Orgeas M Ibn Essaied W Schwebel C Darmon M Mourvillier B. Attributable mortality of ICU-acquired bloodstream infections: impact of the source, causative micro-organism, resistance profile and antimicrobial therapy. J Inf Secur. (2017) 74:131–41. doi: 10.1016/j.jinf.2016.11.001, PMID: 27838521

[3] Delle Rose D Sordillo P Gini S Cerva C Boros S Rezza G. Microbiologic characteristics and predictors of mortality in bloodstream infections in intensive care unit patients: a 1-year, large, prospective surveillance study in 5 Italian hospitals. Am J Infect Control. (2015) 43:1178–83. doi: 10.1016/j.ajic.2015.06.023, PMID: 26253805

[4] Prowle JR Echeverri JE Ligabo EV Sherry N Taori GC Crozier TM. Acquired bloodstream infection in the intensive care unit: incidence and attributable mortality. Crit Care. (2011) 15:R100. doi: 10.1186/cc10114, PMID: 21418635 PMC3219371

[5] Vincent JL Rello J Marshall J Silva E Anzueto A Martin CD. International study of the prevalence and outcomes of infection in intensive care units. JAMA. (2009) 302:2323–9. doi: 10.1001/jama.2009.1754, PMID: 19952319

[6] Timsit JF Ruppé E Barbier F Tabah A Bassetti M. Bloodstream infections in critically ill patients: an expert statement. Intensive Care Med. (2020) 46:266–84. doi: 10.1007/s00134-020-05950-6, PMID: 32047941 PMC7223992

[7] Zhu S Kang Y Wang W Cai L Sun X Zong Z. The clinical impacts and risk factors for non-central line-associated bloodstream infection in 5046 intensive care unit patients: an observational study based on electronic medical records. Crit Care. (2019) 23:52. doi: 10.1186/s13054-019-2353-5, PMID: 30777109 PMC6379966

[8] Sante L Aguirre-Jaime A Miguel MA Ramos MJ Pedroso Y Lecuona M. Epidemiological study of secondary bloodstream infections: the forgotten issue. J Infect Public Health. (2019) 12:37–42. doi: 10.1016/j.jiph.2018.08.011, PMID: 30266540

[9] van Vught LA Klein Klouwenberg PM Spitoni C Scicluna BP Wiewel MA Horn J. Incidence, risk factors, and attributable mortality of secondary infections in the intensive care unit after admission for Sepsis. JAMA. (2016) 315:1469–79. doi: 10.1001/jama.2016.2691 26975785

[10] Tabah A Buetti N Staiquly Q Ruckly S Akova M Aslan AT. Epidemiology and outcomes of hospital-acquired bloodstream infections in intensive care unit patients: the EUROBACT-2 international cohort study. Intensive Care Med. (2023) 49:178–90. doi: 10.1007/s00134-022-06944-2, PMID: 36764959 PMC9916499

[11] Vincent JL Sakr Y Singer M Martin-Loeches I Machado FR Marshall JC. Prevalence and outcomes of infection among patients in intensive care units in 2017. JAMA. (2020) 323:1478–87. doi: 10.1001/jama.2020.2717, PMID: 32207816 PMC7093816

[12] De Waele JJ Akova M Antonelli M Canton R Carlet J De Backer D. Antimicrobial resistance and antibiotic stewardship programs in the ICU: insistence and persistence in the fight against resistance. A position statement from ESICM/ESCMID/WAAAR round table on multi-drug resistance. Intensive Care Med. (2018) 44:189–96. doi: 10.1007/s00134-017-5036-1 29288367

[13] Vincent JL Moreno R Takala J Willatts S De Mendonça A Bruining H. The SOFA (Sepsis-related organ failure assessment) score to describe organ dysfunction/failure. On behalf of the working group on Sepsis-related problems of the European Society of Intensive Care Medicine. Intensive Care Med. (1996) 22:707–10. doi: 10.1007/BF01709751, PMID: 8844239

[14] Rhee JY Kwon KT Ki HK Shin SY Jung DS Chung DR. Scoring systems for prediction of mortality in patients with intensive care unit-acquired sepsis: a comparison of the Pitt bacteremia score and the acute physiology and chronic health evaluation II scoring systems. Shock. (2009) 31:146–50. doi: 10.1097/SHK.0b013e318182f98f, PMID: 18636041

[15] Garner JS Jarvis WR Emori TG Horan TC Hughes JM. CDC definitions for nosocomial infections, 1988. Am J Infect Control. (1988) 16:128–40. doi: 10.1016/0196-6553(88)90053-3, PMID: 2841893

[16] Horan TC Andrus M Dudeck MA. CDC/NHSN surveillance definition of health care-associated infection and criteria for specific types of infections in the acute care setting. Am J Infect Control. (2008) 36:309–32. doi: 10.1016/j.ajic.2008.03.002, PMID: 18538699

[17] Magiorakos AP Srinivasan A Carey RB Carmeli Y Falagas ME Giske CG. Multidrug-resistant, extensively drug-resistant and pandrug-resistant bacteria: an international expert proposal for interim standard definitions for acquired resistance. Clin Microbiol Infect. (2012) 18:268–81. doi: 10.1111/j.1469-0691.2011.03570.x, PMID: 21793988

[18] Chattaway MA Schaefer U Tewolde R Dallman TJ Jenkins C. Identification of Escherichia coli and Shigella species from whole-genome sequences. J Clin Microbiol. (2017) 55:616–23. doi: 10.1128/JCM.01790-16, PMID: 27974538 PMC5277532

[19] Warren MA Zhao Z Koyama T Bastarache JA Shaver CM Semler MW. Severity scoring of lung oedema on the chest radiograph is associated with clinical outcomes in ARDS. Thorax. (2018) 73:840–6. doi: 10.1136/thoraxjnl-2017-211280, PMID: 29903755 PMC6410734

[20] O'Grady NP Alexander M Burns LA Dellinger EP Garland J Heard SO. Summary of recommendations: guidelines for the prevention of intravascular catheter-related infections. Clin Infect Dis. (2011) 52:1087–99. doi: 10.1093/cid/cir138, PMID: 21467014 PMC3106267

[21] Teng SO Yen MY Ou TY Chen FL Yu FL Lee WS. Comparison of pneumonia- and non-pneumonia-related *Acinetobacter baumannii* bacteremia: impact on empiric therapy and antibiotic resistance. J Microbiol Immunol Infect. (2015) 48:525–30. doi: 10.1016/j.jmii.2014.06.011, PMID: 25103719

[22] Xu J Xu Y Zheng X. Comparison of pneumonia and nonpneumonia-related *Acinetobacter baumannii* complex bacteremia: a single-center retrospective study. Am J Infect Control. (2023) 51:567–73. doi: 10.1016/j.ajic.2022.08.004, PMID: 35948125

[23] Tabah A Koulenti D Laupland K Misset B Valles J Bruzzi de Carvalho F. Characteristics and determinants of outcome of hospital-acquired bloodstream infections in intensive care units: the EUROBACT international cohort study. Intensive Care Med. (2012) 38:1930–45. doi: 10.1007/s00134-012-2695-9 23011531

[24] Kallel H Houcke S Resiere D Roy M Mayence C Mathien C. Epidemiology and prognosis of intensive care unit-acquired bloodstream infection. Am J Trop Med Hyg. (2020) 103:508–14. doi: 10.4269/ajtmh.19-0877, PMID: 32314689 PMC7356483

[25] Gouel-Cheron A Swihart BJ Warner S Mathew L Strich JR Mancera A. Epidemiology of ICU-onset bloodstream infection: prevalence, pathogens, and risk factors among 150,948 ICU patients at 85 U.S. Hosp Crit Care Med. (2022) 50:1725–36. doi: 10.1097/CCM.0000000000005662 PMC10829879 36190259

[26] Au-Yong I Higashi Y Giannotti E Fogarty A Morling JR Grainge M. Chest radiograph scoring alone or combined with other risk scores for predicting outcomes in COVID-19. Radiology. (2022) 302:460–9. doi: 10.1148/radiol.2021210986, PMID: 34519573 PMC8475750

[27] van der Zee P Gommers D. Recruitment maneuvers and higher PEEP, the so-called open lung concept, in patients with ARDS. Crit Care. (2019) 23:73. doi: 10.1186/s13054-019-2365-1, PMID: 30850004 PMC6408810

[28] Papazian L Munshi L Guérin C. Prone position in mechanically ventilated patients. Intensive Care Med. (2022) 48:1062–5. doi: 10.1007/s00134-022-06731-z, PMID: 35652920 PMC9160174

[29] Langer T Brioni M Guzzardella A Carlesso E Cabrini L Castelli G. Prone position in intubated, mechanically ventilated patients with COVID-19: a multi-centric study of more than 1000 patients. Crit Care. (2021) 25:128. doi: 10.1186/s13054-021-03552-2, PMID: 33823862 PMC8022297

[30] Richard JC Sigaud F Gaillet M Orkisz M Bayat S Roux E. Response to PEEP in COVID-19 ARDS patients with and without extracorporeal membrane oxygenation. A multicenter case-control computed tomography study. Crit Care. (2022) 26:195. doi: 10.1186/s13054-022-04076-z 35780154 PMC9250720

[31] Holmes CL Anderson MT Mobley HLT Bachman MA. Pathogenesis of gram-negative bacteremia. Clin Microbiol Rev. (2021) 34:e00234–20. doi: 10.1128/CMR.00234-20, PMID: 33692149 PMC8549824

[32] Orsi GB Giuliano S Franchi C Ciorba V Protano C Giordano A. Changed epidemiology of ICU acquired bloodstream infections over 12 years in an Italian teaching hospital. Minerva Anestesiol. (2015) 81:980–8. PMID: 25411769

[33] Xie J Li S Xue M Yang C Huang Y Chihade DB. Early- and late-onset bloodstream infections in the intensive care unit: a retrospective 5-year study of patients at a University Hospital in China. J Infect Dis. (2020) 221:S184–92. doi: 10.1093/infdis/jiz606 32176791

[34] Haug JB Berild D Walberg M Reikvam A. Increased antibiotic use in Norwegian hospitals despite a low antibiotic resistance rate. J Antimicrob Chemother. (2011) 66:2643–6. doi: 10.1093/jac/dkr361, PMID: 21903657

[35] Kadri SS Adjemian J Lai YL Spaulding AB Ricotta E Prevots DR. Difficult-to-treat resistance in gram-negative bacteremia at 173 US hospitals: retrospective cohort analysis of prevalence, predictors, and outcome of resistance to all first-line agents. Clin Infect Dis. (2018) 67:1803–14. doi: 10.1093/cid/ciy378 30052813 PMC6260171

[36] Huh K Chung DR Ha YE Ko JH Kim SH Kim MJ. Impact of difficult-to-treat resistance in gram-negative bacteremia on mortality: retrospective analysis of Nationwide surveillance data. Clin Infect Dis. (2020) 71:e487–96. doi: 10.1093/cid/ciaa084, PMID: 31994704

