# Supplementary material for: Clinical characteristics and prognosis of pneumonia-related bloodstream infections in the intensive care unit: a single-center retrospective study
Source: Front Public Health. 2023 Sep 8;11:1249695. doi: 10.3389/fpubh.2023.1249695 (PMC10516289; doi:10.3389/fpubh.2023.1249695)
Supplement: Supplementary file 1 [file Data_Sheet_1.zip › Supplementary Figure Legends.docx]

Supplementary Material

1. **Supplementary Figure Legends**

**Supplementary Figure 1.** Consolidation and density scoring in the Radiographic Assessment of Lung Edema (RALE) score

**Supplementary Figure 2.** Phylogenetic tree constructed using WGS of 44 paired pathogen strains from clinically considered PRBSI, including strains isolated from respiratory tract and blood and strains screened from the National Center for Biotechnology Information. (A. *Pseudomonas aeruginosa*, B. *Acinetobacter baumannii*, C. *Klebsiella pneumoniae*, D. *Burkholderia multivorans*, E. *Burkholderia cenocepacia*, F. *Escherichia coli*, G *Morganella morganii*, H *Enterococcus faecium*, I. *Staphylococcus aureus*)

**Supplementary Figure 3.** The curve of laboratory indicators related to infection during the period from ICU admission, 7 days before BSI, 72 hours before BSI, 48 hours before BSI, and 24 hours before BSI to BSI onset. (A. White blood cell count, B. Neutrophil count, C. Platelet count, D. Lactic acid, E. Procalcitonin, F. D-dimer)

**Supplementary Figure 4.** Distribution of pathogens of PRBSI
