# Supplementary material for: Clinical characteristics and prognosis of pneumonia-related bloodstream infections in the intensive care unit: a single-center retrospective study
Source: Front Public Health. 2023 Sep 8;11:1249695. doi: 10.3389/fpubh.2023.1249695 (PMC10516289; doi:10.3389/fpubh.2023.1249695)
Supplement: Supplementary file 1 [file Data_Sheet_1.zip › Supplementary Tables.docx]

Supplementary Material

1. **Supplementary Tables**

| **Supplementary Table 1. Source of all ICU bloodstream infections** | |  |
| --- | --- | --- |
|  |  |  |
| **Source** | **no. (%)** |  |
| **Primary BSI** | 37 (30.8) |  |
| **Pneumonia** | 32 (26.7) |  |
| **Intravascular catheter** | 28 (23.3) |  |
| **Intra‐abdominal tract** | 12 (10.0) |  |
| **Urinary tract** | 4 (3.3) |  |
| **Skin and soft tissues** | 4 (3.3) |  |
| **Endocarditis** | 3 (2.5) |  |

BSI: bloodstream infection.

| **Supplementary Table 2. Laboratory indicators related to infection analysis between PRBSI and non-PRBSI groups** | | | | |  |
| --- | --- | --- | --- | --- | --- |
|  |  |  |  |  |  |
| **Characteristics** | **Total BSI n=120** | **PRBSI n=32** | **Non-PRBSI n=88** | ***p* value** |  |
| **White blood cell count, *10^9^/L** |  |  |  |  |  |
| ICU admission | 11.43 (8.02, 16.32) | 9.20 (6.36, 12.19) | 12.64 (9.36, 17.66) | **0.021** |  |
| 7 days before BSI | 9.97 (6.80, 13.28) | 8.61 (5.81, 13.13) | 10.25 (7.35, 13.59) | 0.317 |  |
| 72h before BSI | 10.67 (6.61, 14,67) | 7.94 (5.92, 14.32) | 11.05 (8.24, 15.06) | 0.254 |  |
| 48h before BSI | 10.85 (6.46, 16.02) | 9.93 (5.73, 15.77) | 11.29 (6.94, 16.71) | 0.507 |  |
| 24h before BSI | 11.23 (6.46, 17.40) | 10.63 (6.16, 14.62) | 12.03 (6.89, 17.59) | 0.380 |  |
| BSI onset | 11.35 (6.74, 17.96) | 10.93 (5.26, 17.81) | 11.52 (7.24, 17.96) | 0.502 |  |
| **Neutrophil count, *10^9^/L** |  |  |  |  |  |
| ICU admission | 10.45 (6.88, 15.41) | 7.79 (5.59, 11.48) | 11.44 (8.87, 16.00) | **0.019** |  |
| 7 days before BSI | 8.16 (5.97, 12.29) | 7.68 (5.13, 11.92) | 9.32 (6.11, 12.32) | 0.415 |  |
| 72h before BSI | 9.77 (5.55, 13.30) | 6.54 (5.23, 12.96) | 9.93 (7.24, 13.34) | 0.171 |  |
| 48h before BSI | 9.58 (5.43, 14.50) | 8.62 (4.98, 12.78) | 9.89 (6.34, 15.29) | 0.373 |  |
| 24h before BSI | 10.28 (4.97, 16.33) | 8.83 (5.01, 13.16) | 10.45 (5.30, 16.56) | 0.473 |  |
| BSI onset | 10.28 (5.83, 16.05) | 9.63 (4.03, 16.22) | 10.46 (6.39, 15.96) | 0.481 |  |
| **Platelet count, *10^9^/L** |  |  |  |  |  |
| ICU admission | 141.50 (73.75, 209.25) | 150.00 (86.50, 208.50) | 130.50 (70.00, 216.75) | 0.484 |  |
| 7 days before BSI | 106.00 (64.25, 171.25) | 119.00 (66.00, 191.00) | 95.00 (63.50, 155.00) | 0.255 |  |
| 72h before BSI | 91.00 (60.00, 175.50) | 89.00 (61.50, 144.00) | 101.50 (60.00, 181.50) | 0.570 |  |
| 48h before BSI | 104.50 (63.00, 162.50) | 96.00 (62.25, 150.00) | 109.50 (64.25, 163.00) | 0.359 |  |
| 24h before BSI | 97.00 (57.00, 161.00) | 82.00 (46.00, 177.00) | 114.50 (63.00, 157.25) | 0.747 |  |
| BSI onset | 90.00 (50.75, 164.00) | 82.00 (35.75, 180.50) | 90.50 (52.75, 158.75) | 0.620 |  |
| **Lactic acid, mmol/L** |  |  |  |  |  |
| ICU admission | 1.90 (1.30, 2.70) | 1.80 (1.30, 2.52) | 1.90 (1.30, 2.75) | 0.888 |  |
| 7 days before BSI | 1.75 (1.25, 2.50) | 1.80 (1.40, 2.70) | 1.70 (1.20, 2.30) | 0.483 |  |
| 72h before BSI | 1.80 (1.20, 2.30) | 1.70 (1.35, 2.15) | 1.80 (1.15, 2.45) | 0.913 |  |
| 48h before BSI | 1.70 (1.42, 2.65) | 1.65 (1.50, 2.85) | 1.75 (1.37, 2.50) | 0.646 |  |
| 24h before BSI | 1.90 (1.30, 2.60) | 2.10 (1.55, 2.55) | 1.80 (1.20, 2.58) | 0.234 |  |
| BSI onset | 2.55 (1.60, 4.07) | 2.60 (1.70, 3.50) | 2.40 (1.52, 4.07) | 0.639 |  |
| **Procalcitonin, mg/L** |  |  |  |  |  |
| ICU admission | 1.41 (0.67, 7.74) | 1.29 (0.69, 8.9) | 1.43 (0.58 -7.70) | 0.610 |  |
| 7 days before BSI | 0.76 (0.38, 2.65) | 0.61 (0.38, 2.18) | 0.96 (0.45, 2.88) | 0.627 |  |
| 72h before BSI | 1.24 (0.46, 3.87) | 1.51 (0.43, 5.24) | 1.12 (0.51, 3.67) | 0.712 |  |
| 48h before BSI | 1.24 (0.53, 4.71) | 1.42 (0.40, 4.48) | 1.24 (0.56, 4.90) | 0.980 |  |
| 24h before BSI | 1.81 (0.58, 7.54) | 1.23 (0.37, 6.31) | 1.98 (0.72, 7.62) | 0.278 |  |
| BSI onset | 4.22 (1.22, 11.22) | 2.63 (0.96, 7.70) | 5.25 (1.33, 13.53) | 0.072 |  |
| **D-dimer, mg/L** |  |  |  |  |  |
| ICU admission | 5.38 (2.52, 8.58) | 5.27 (2.84, 7.58) | 5.41 (2.52, 9.65) | 0.948 |  |
| 7 days before BSI | 5.11 (2.75, 9.31) | 4.95 (2.90, 9.08) | 5.68 (2.36, 14.35) | 0.835 |  |
| 72h before BSI | 4.43 (2.56, 9.62) | 4.44 (2.42, 9.24) | 4.41 (2.70, 15.42) | 0.534 |  |
| 48h before BSI | 4.39 (2.77, 11.92) | 4.15 (3.16, 6.63) | 5.70 (2.60, 14.61) | 0.558 |  |
| 24h before BSI | 4.51 (2.71, 12.51) | 4.51 (2.75, 12.30) | 4.51 (2.70, 12.22) | 0.943 |  |
| BSI onset | 3.96 (2.37, 8.38) | 3.12 (2.23, 7.06) | 4.21 (2.62, 9.81) | 0.206 |  |
| All continuous data are given as median (IQR)  BSI: bloodstream infection. | | | | |  |
