# Supplementary figures and images for: Clinical characteristics and prognosis of pneumonia-related bloodstream infections in the intensive care unit: a single-center retrospective study
Source: Front Public Health. 2023 Sep 8;11:1249695. doi: 10.3389/fpubh.2023.1249695 (PMC10516289; doi:10.3389/fpubh.2023.1249695)

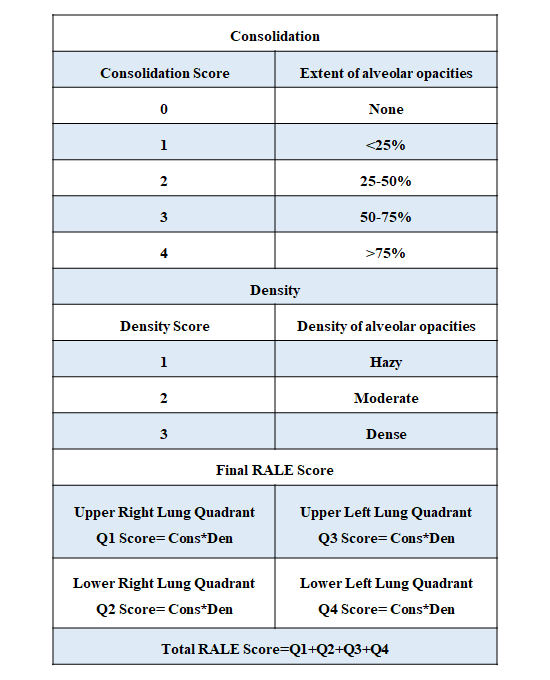

Supplement: Supplementary file 1 [file Data_Sheet_1.zip › Supplementary Figure 1.TIF]

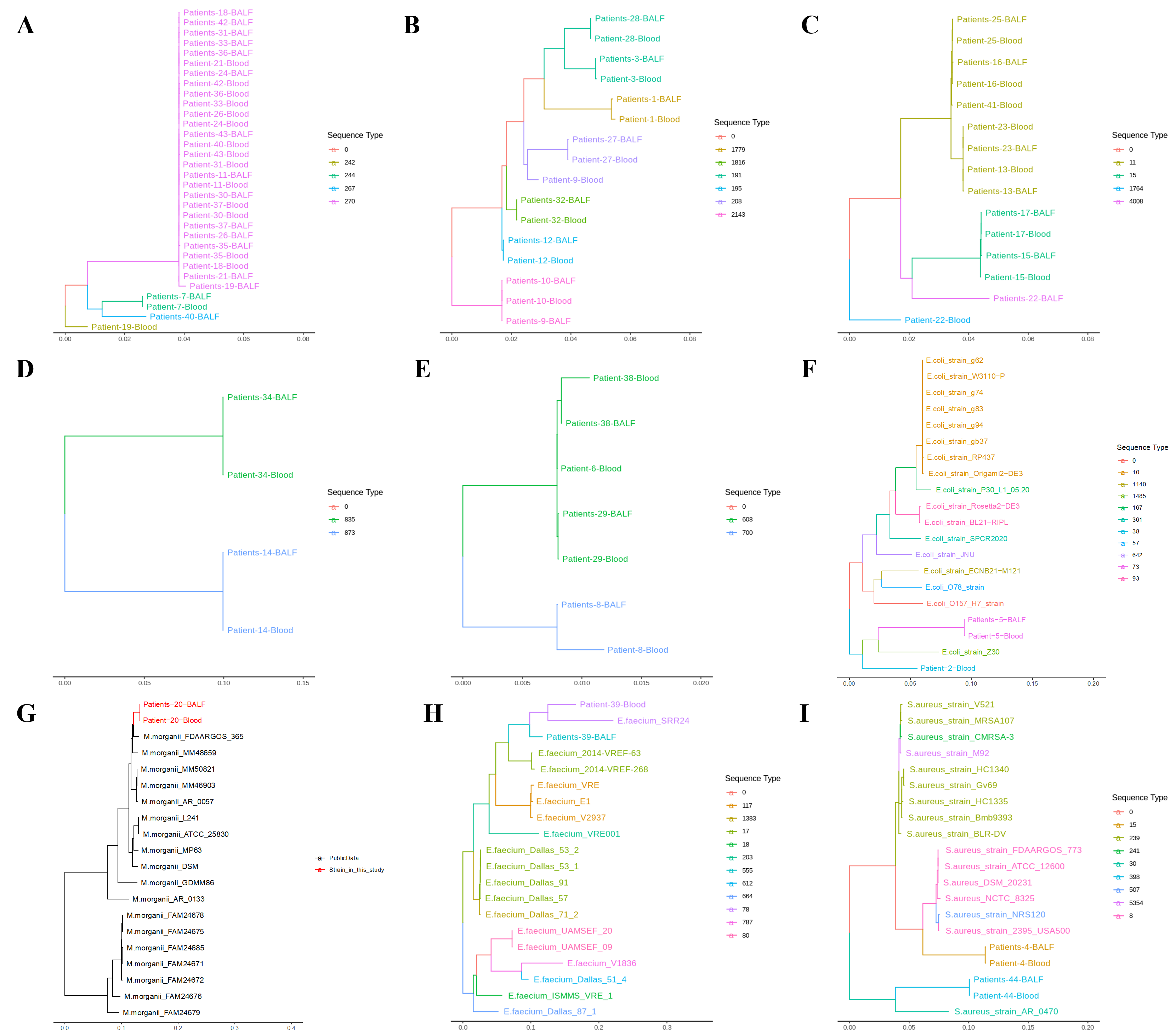

Supplement: Supplementary file 1 [file Data_Sheet_1.zip › Supplementary Figure 2.TIF]

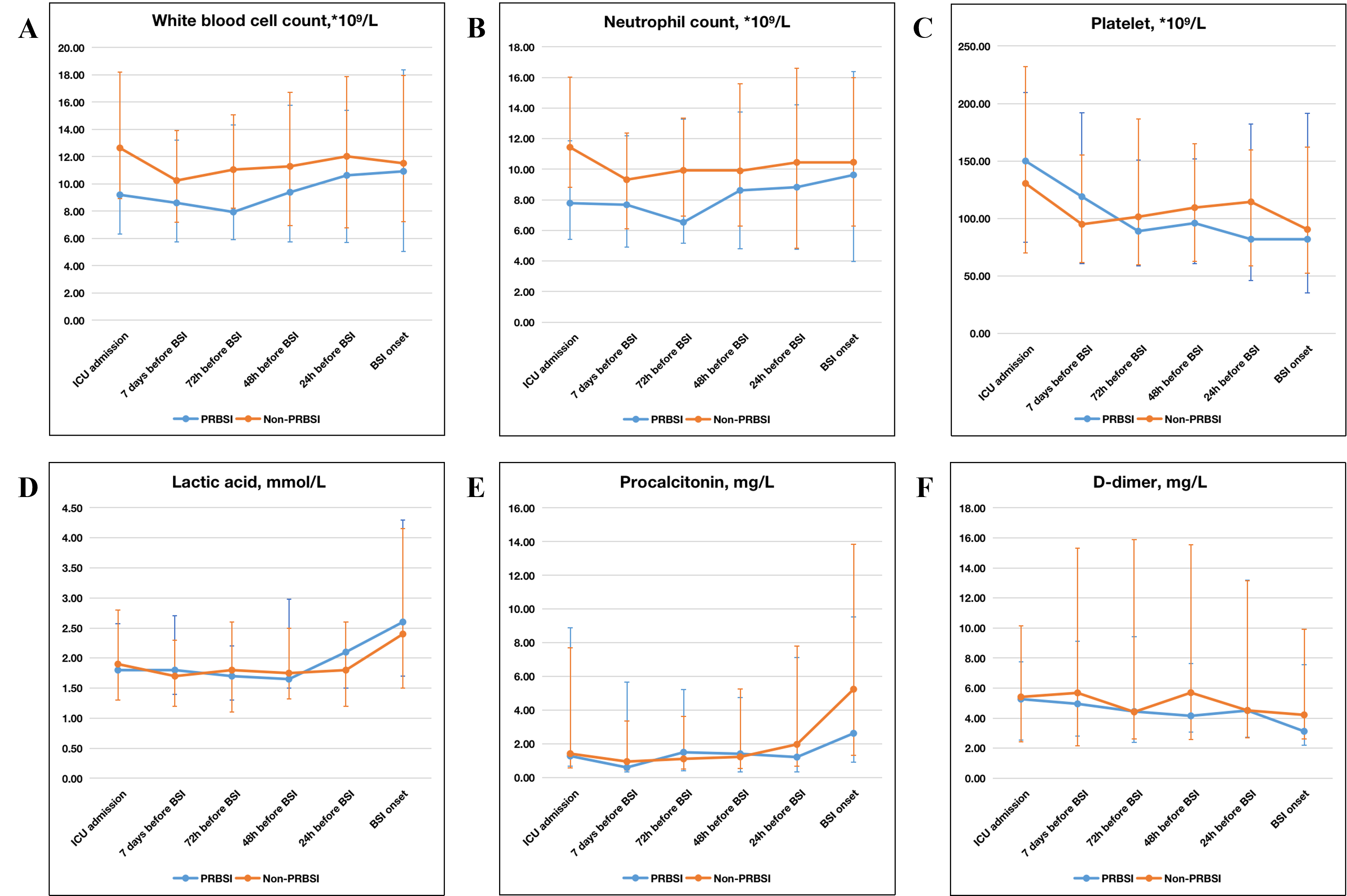

Supplement: Supplementary file 1 [file Data_Sheet_1.zip › Supplementary Figure 3.TIF]

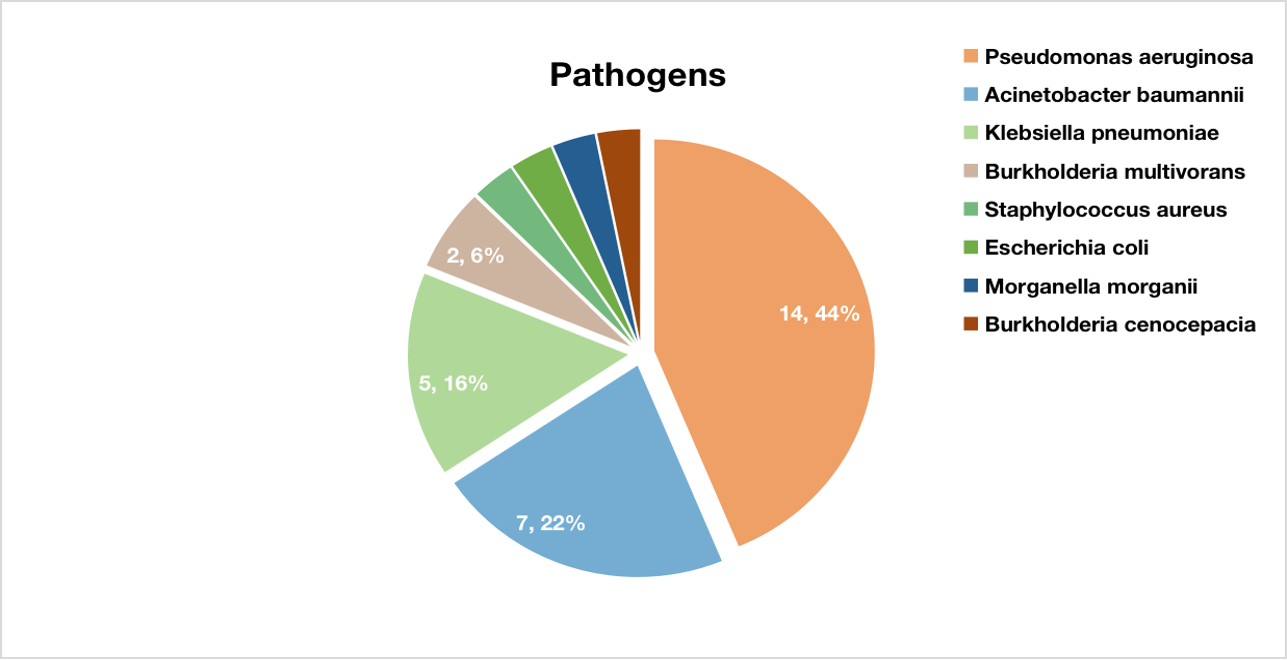

Supplement: Supplementary file 1 [file Data_Sheet_1.zip › Supplementary Figure 4.TIF]
